# Deep learning-based robust automatic non-invasive measurement of blood pressure using Korotkoff sounds

**DOI:** 10.1038/s41598-021-02513-7

**Published:** 2021-12-03

**Authors:** Ji-Ho Chang, Il Doh

**Affiliations:** 1 Division of Physical Metrology, Korea Research Institute of Standards and Science (KRISS), Daejeon, Republic of Korea; 2 Safety Measurement Institute, Korea Research Institute of Standards and Science (KRISS), Daejeon, Republic of Korea; 3 Department of Medical Physics, University of Science and Technology (UST), Daejeon, Republic of Korea

**Keywords:** Biomedical engineering, Physical examination

## Abstract

This paper proposes a method that automatically measures non-invasive blood pressure (BP) based on an auscultatory approach using Korotkoff sounds (K-sounds). There have been methods utilizing K-sounds that were more accurate in general than those using cuff pressure signals only under well-controlled environments, but most were vulnerable to the measurement conditions and to external noise because blood pressure is simply determined based on threshold values in the sound signal. The proposed method enables robust and precise BP measurements by evaluating the probability that each sound pulse is an audible K-sound based on a deep learning using a convolutional neural network (CNN). Instead of classifying sound pulses into two categories, audible K-sounds and others, the proposed CNN model outputs probability values. These values in a Korotkoff cycle are arranged in time order, and the blood pressure is determined. The proposed method was tested with a dataset acquired in practice that occasionally contains considerable noise, which can degrade the performance of the threshold-based methods. The results demonstrate that the proposed method outperforms a previously reported CNN-based classification method using K-sounds. With larger amounts of various types of data, the proposed method can potentially achieve more precise and robust results.

## Introduction

Blood pressure (BP) is one of the primary vital signs in the human body and is one of the most important physical quantities, not only for a clinical diagnosis of hypertension but also for personal healthcare. Although the most accurate BP measurement method is to insert a pressure probe into an artery, this invasive method is limited in use due to the high risk of bleeding and infection. Thus, non-invasive blood pressure (NIBP) measurement methods have been proposed, such as the auscultatory and the oscillometric methods. When using the auscultatory method, a cuff is wrapped around the subject’s upper limb and an observer listens to the Korotkoff sounds (K-sounds) using a stethoscope. This method has served as the gold standard for BP measurements for more than 100 years in clinics^1^. Nonetheless, a well-trained observer is necessary for this method, which motivates the development of the oscillometric method. This method measures the BP by analyzing cuff pressure oscillations without any observer, and it has been applied to automated BP monitoring devices. The cuff pressure oscillations are caused by the pressure difference between internal artery pressure and surrounding cuff pressure. However, because this oscillometric method does not use K-sounds, the results inherently demonstrate considerable differences from those by the auscultatory method. Even with an automated BP device that had passed a standardized validation assessment (ISO 81060-2:2018)^2^, the difference occasionally reaches 20 mmHg^3^, which is unsatisfactory considering that a 5 mmHg error can double or halve the number of hypertensive patients^4^.

To improve the accuracy of BP measurements, automated methods using K-sounds have been proposed^5–11^. Rather than the cuff pressure oscillation, K-sounds was recorded by a microphone attached to skin and analyzed to determine BPs. The results demonstrated small deviations from the auscultatory reference under a well-controlled environment. However, most of these methods are based on the threshold of the sound pressure from a stethoscope. That is, the cuff pressure when the sound pressure exceeds the threshold is determined as the systolic blood pressure (SBP), and the cuff pressure when the sound pressure decreases below the threshold is the diastolic blood pressure (DBP). These methods are vulnerable to the measurement conditions and external noise, and the results can therefore be degraded in actual BP measurement environments. One study proposed a method robust to external noise, but two microphones were necessary, and validation for subjects under various noisy environments is required^8^.

Recently, a deep learning-based method that uses acoustic signals was proposed^12^. In this method, a convolutional neural network (CNN) model was used to classify the sound signals as K-sounds or other sounds. When two consecutive pulses were assessed as K-sounds initially, the cuff pressure at the first pulse was regarded as the SBP. When no additional K-sounds appeared, the cuff pressure at the moment was the DBP. This study demonstrated that a deep learning method could precisely detect K-sounds in spite of the difference in the contact pressure between the microphone and the skin. However, the reference SBP and DBP values were from playback of the recorded audio, which was very likely to differ from the values from observers using a stethoscope. More importantly, there was no decision rule for the determination of BPs from the classified K-sounds^12^ and they were determined individually. Accurate decision of BPs from the classified K-sounds was very vulnerable to various external noises and, there was no robust decision rule so far.

The present study proposes a deep learning-based automated measurement method using K-sounds, yet the present study aimed to improve the performance and the robustness by taking the human response to K-sounds into consideration. A curve presumed that reflected the human response was utilized to assign a probability value to each pulse, and was then applied to determine the SBP and DBP. In addition, augmentation methods were employed to prevent the CNN model from over-fitting. Each pulse was converted into an image of multiple band-pass-filtered signals instead of spectrograms to increase the frequency resolution and to contain the phase information.

For validation of this method, 277 measurement sets from 43 subjects were collected in practice, meaning that various noise signals were included. These sets contain 10,743 pulses in total. These datasets were split into the training set, the validation set, and the test set to apply hold-out validation^13^. The test set was prepared to meet the conditions for a standardized validation as much as possible^2^. The CNN was trained with the training set, and the estimated BP values for the test set were compared with other methods, i.e., the auscultatory method and a deep learning-based classification method.

## Background

### BP measurement by the auscultatory method

When the cuff is pressurized above the BP, the artery is collapsed and no blood can flow. When the cuff pressure decreases below the SBP, blood starts to overcome the cuff pressure and flow intermittently. That is, when the cuff pressure is deflated, the cuff signal fluctuates with the blood pulses, which induces local peaks in the cuff pressure as well as the sound signal (K-sounds). K-sounds are likely produced by harmonic oscillations of the artery wall, acting as a spring-mass-damper, as it closes and opens with each blood pulse when the cuff pressure is between SBP and DBP. When the cuff pressure is lowered to the DBP, blood flows without any obstruction and the Korotkoff sounds disappear. When using this method, an observer listens to the K-sounds using a stethoscope to find the critical cuff pressure values at the moment the K-sounds appear and disappear, which correspond to SBP and DBP, respectively.

The spectral characteristics of K-sounds have long been studied^14–18^, and the effects of several parameters on BP measurements and the variations were recently investigated^19,20^. These studies demonstrated that the spectral characteristics differs from person to person and considerably depend on the measurement conditions and the phase of the K-sounds. This implies some difficulty in deriving an analytical model for K-sounds.

### Automated BP measurement by the oscillometric method

**Figure 1:**
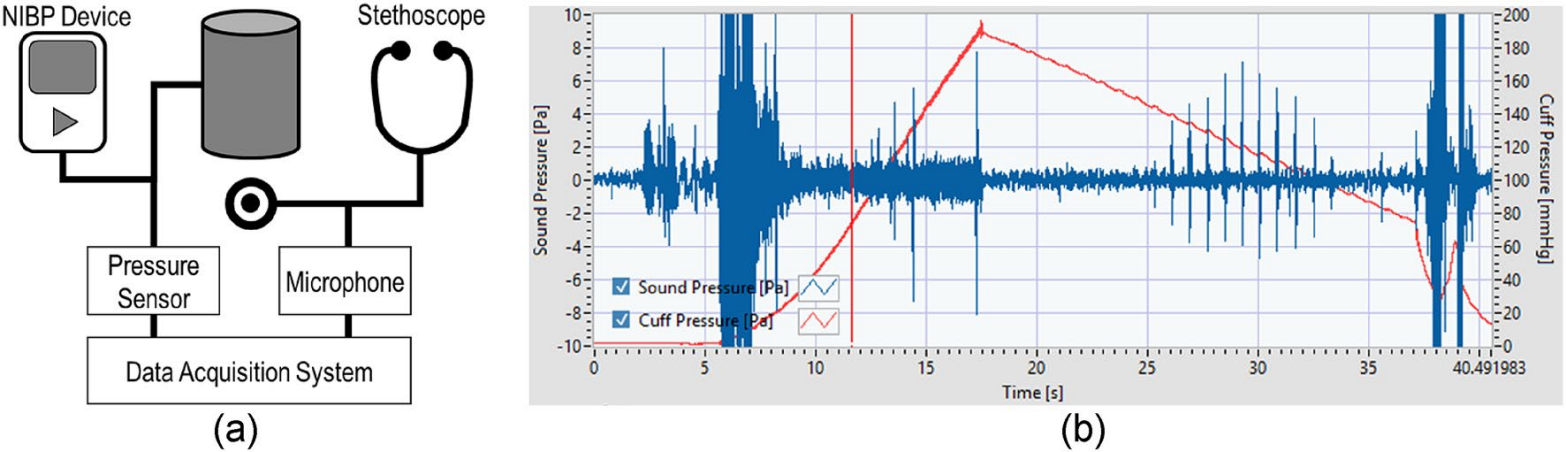
Blood pressure data collection system using a stethoscope and data acquisition board: (**a**) data acquisition system and stethoscope connected to a microphone, and (**b**) LabVIEW-based data acquisition software.

As the cuff pressure deflates, a small amount of pressure oscillation (typically less than 3 mmHg) is observed due to the intermittent blood flow in the artery. When the using oscillometric method for BP measurements, this cuff pressure oscillation, which overlaps the overall decrease of the cuff pressure, is extracted and analyzed. Unlike the auscultatory method, there are no critical points for the determination of the SBP and DBP because the oscillation appears even when the cuff pressure is beyond the range between SBP and DBP. Hence, the cuff pressure oscillation amplitude is analyzed to estimate the auscultatory SBP and DBP. In amplitude-based algorithms, the SBP and DBP are determined relative to the maximum amplitude, as shown in Fig. 1b. Otherwise, derivative algorithms determine the points at which the slope of the oscillogram becomes the maximum and minimum of the SBP and DBP, respectively^21^. Although this method has been widely applied to automated BP measurements because no observer is required for K-sounds, the accuracy is inherently low as K-sounds are not utilized.

### Automated BP measurement based on the K-sounds

To overcome the limitations of the oscillometric method, K-sounds have been used for automated BP measurements. When using these methods, a microphone is installed onto a stethoscope, which is placed under the cuff on the upper arm, and sound from the artery is recorded. The SBP and DBP are determined by detecting the K-sounds.

#### Threshold based methods

Most of these methods are based on the thresholds of processed values from acoustic signals, showing good results under well-controlled conditions. For example, Regueiro-Gomez and Pallas-Areny defined an indicator called the ratio of spectral energy dispersion (RSED) and detected peaks that have the maximum RSED^7^. Sebald et al. calculated intensity of individual pulses, and 50% and 30% of the plateau were determined correspondingly as the SBP and the DBP^8^. Park et al. determined SBP and DBP points when there was an abrupt jump or drop in the total power values of the pulses; the resultant mean and standard deviation of the difference from the auscultation method were 2.0 ± 3.3 (mean ± standard deviation) and 3.1 ± 4.0 mmHg for the SBP and DBP, respectively^9^. In other work^10^, all peaks that exceed an amplitude threshold seem to be determined as audible K-sounds, although it is not clear because the decision rule was not stated in detail. The results were 1.2 ± 1.7 mmHg for the SBP error and 0.7 ± 1.2 mmHg for the DBP error for 45 measurements (15 subjects × 3 times). Hong et al. applied a human hearing threshold; each peak is filtered to four octave frequency bands that contains the main components of the K-sounds (center frequency: 31.5, 63, 128, and 250 Hz), and when the magnitude in at least three bands exceed the hearing threshold, the peak is assessed as an audible K-sound. The mean differences in the SBP and DBP were 0.3 ± 2.0 and 1.2 ± 2.2 mmHg, respectively^11^.

However, if external noise is recorded together with the K-sound, the performance can be significantly degraded because noise signals before the SBP or after the DBP can exceed the threshold, and accordingly distort the SBP and the DBP values.

#### Deep learning-based method

Deep learning-based methods can be good alternatives to determine BPs. Deep learning had shown better or at least comparable performance to humans in many applications, such as vision and signal processing. Although it has chance to show incorrect results due to an inadequate training^22^, deep learning has the potential to differentiate K-sounds and noise signals using the characteristics of the signals, as humans do.

Pan et al. utilized a deep learning method to analyze variations in K-sounds^20^. Their results showed that K-sounds could be consistently identified during the period between the SBP and the DBP, whereas there can be considerable variations in the systolic and diastolic components.

In addition, these authors proposed a deep learning-based method that utilizes K-sounds^12^. A simple CNN was designed and trained to identify the K-sounds. The effects of the stethoscope position and the contact pressure on the measured BPs were found to be insignificant, implying that their deep learning-based method can successfully identify K-sounds in spite of the differences in certain measurement conditions. As the decision rule, when at least two consecutive pulses are determined as K-sounds, the cuff pressure at the first pulse is obtained as the SBP. The DBP is ascertained when no pulse is assessed as the K-sound. The mean differences between the proposed method and the auscultatory method are 1.4 ± 2.4 and 3.3 ± 2.9 mmHg for the SBP and DBP, respectively. However, this decision rule is also vulnerable to external noise; moreover, as mentioned in “Introduction”, there were too few subjects. Furthermore, the BPs through the auscultatory method were not determined according to a standard protocol^2^, and inconsistencies at the systole and diastole components were not considered in the design of the deep learning model.

In the present study, a model similar to that proposed in earlier work^12^ is employed as a baseline model. The details are given in “Proposed method”.

### Fundamentals of the CNN

The CNN was initially proposed for processing images, speech and time series^23^. Unlike traditional models of pattern recognition in which hand-designed feature extractors obtain relevant information from the input, the CNN optimizes feature extractors when training the model. These feature extractors are termed filters or kernels. Through nonlinear processing in several layers, the feature maps are finally mapped into multiple independent neurons of the last layer, which are then linked to probabilities of specific classes to which the inputs belong. Several CNN models such as VGGnet, Resnet, Densenet, and efficient net, have been designed^24–28^ and the performance capabilities have been shown to increase in general as the number of layers and the numbers of neurons increase^24^.

To determine whether or not an acoustic signal contains K-sounds, a simple way to design the CNN model would be to sort the classes into two categories: K-sound and others, as in earlier work^12^. However, because there are substantial uncertainties in the systole and diastole components^20^, this strategy can lead to inconsistent training datasets with the model, which can then affect the performance. Alternatively, a CNN model can be designed to output a probability value between 0 and 1; when this value approaches 1, the signal is more likely to contain K-sounds.

## Data acquisition and proposed method

In the present study, a CNN model that outputs a probability value was designed. To train the model, pulses at the systole or diastole phases were labeled with a probability value between 0 and 1 based on a curve that reflects the human response, aiming to take the human response in consideration. Data acquisition for this study is described in the first subsection, and details of the proposed methods are given in the following subsections.

### Data acquisition

From 42 healthy subjects aged 20 to 60, the cuff and the microphone signals were recorded. This study was approved by the Institutional Review Board of KRISS (IRB 200428-04) and informed consent forms were obtained from the subjects. All methods were performed in accordance with the relevant guidelines and regulations. A commercially available NIBP device was used to control the cuff pressure for the BP measurements. Reference BP measurement was performed in accordance with the ISO 81060-2^2^. Two observers listened to Korotkoff sounds using a double stethoscope to measure the BPs independently. Their average BP values were used as the reference BPs only if the difference was within 4 mmHg. To obtain cuff pressure and K-sound, a pressure sensor (UNIK5000) and a microphone (GLAS 46BL) were used, respectively. The cuff pressure was measured to extract oscillometric waveform and obtain time frame for K-sounds acquisition. Like conventional auscultatory blood pressure measurement, chest piece of the stethoscope was placed outside the cuff over the brachial artery. And the contact force between chest piece of stethoscope and skin was adjusted using a stretchable band so that the diaphragm was in contact with the skin and did not press excessively. The diaphragm of the chest piece was facing the skin and confirmed that the head was turned so that there was an open-air passage between the diaphragm and connecting tubing. As shown in Fig. 1, a 24 bit data acquisition board (NI 9232) was used to collect the signals from each sensor. The sampling frequency was 2560 Hz for both the cuff pressure and the sound pressure.

The measurement was conducted from April to September of 2020. The total numbers of subjects, Korotkoff cycles, and beats are presented in Table 1. The beats generated by periodic motion of heart were recognized from the cuff pressure, and Korotkoff cycle was the sound signals at the time frame of each beat between SBP and DBP. Without the time frame for K-sound, training for CNN would be very inefficient. All segments were divided into the training set, validation set, and test set. Data from 10 subjects were included both in the training and test sets, which was admittedly inevitable due to the limited number of subjects. However, the data in the test set were not included in the training set. Data from the subject wearing various clothing with sleeves was also collected to diverse the training data. Data for which the SBP or DBP values by two subjects have a difference exceeding 4 mmHg were excluded for the test set, though they were still used for training by taking the mean value of the two. For the test set, we attempted to match the blood pressure distribution in an earlier study, but admittedly we were not able to acquire data for which the SBP exceeded 160 mmHg. The other distributions were matched, as presented in Table 2.

**Table 1:** Number of subjects, Korotkoff cycles, and beats.

	Number of subjects	Number of Korotkoff cycles	Number of beats
Training and validation set	33	217	7,892 (training) + 456 (validation)
Test set	20	60	2,395
Total	43 (10 in common)	277	10,743

**Table 2:** Blood pressure distribution in the test set.

SBP	Number in test set (%)	DBP	Number in test set (%)
≤ 100 (5%)	7 (11.7%)	≤ 60 (5%)	5 (8.3%)
≤ 140 (20%)	12 (20.0%)	≤ 85 (20%)	12 (20.0%)
≥ 160 (5%)	0 (0.0%)	≤ 100 (5%)	4 (6.7%)

### Preprocessing

**Figure 2:**
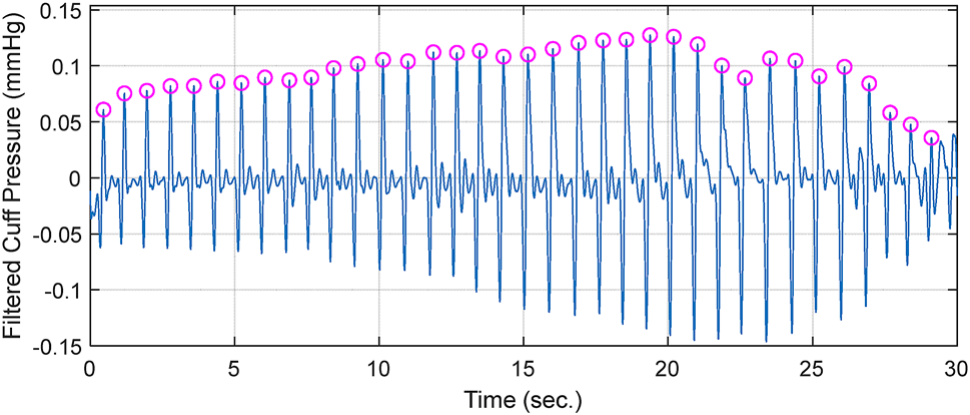
Cuff pressure oscillation for peak extraction.

Sound pressure signals were acquired by a microphone mounted on a stethoscope. Local peaks in the microphone signals were extracted according to the local peaks in the cuff signal. When the cuff pressure was deflated, the cuff signal fluctuated with the blood pulses, which induced local peaks in the cuff signal as well as the sound signal. In this study, the local peaks in the cuff signal were detected after high-pass filtering at 4 Hz and low-pass filtering at 6.67 Hz, corresponding to the maximum blood pulse of 400 divided by 60 s. A time segment that was 0.4 s long, centered at a local peak in the cuff pressure signal was extracted. 0.4 s was optimal length of time frame to obtain K-sound. Shorter times resulted in partial loss of the sound signal, and longer times reduced computational efficiency. Figure 2 shows an example of a cuff pressure oscillation for peak extraction. The detected peaks are marked as circles.

In many deep learning methods, time signals are often transformed into spectrograms by the short-time Fourier transform (STFT). However, because the length of each segment is short and most of the energy of the K-sounds is concentrated at a low frequency, the frequency resolution of spectrograms may be too low. For example, if the length of each frame is 0.04 s, the frequency resolution is 25 Hz. Thus, in the present study, we used band-pass-filtered signals. That is, we designed 50 band-pass filters with a 10 Hz bandwidth with a low cutoff frequency from 25 to 515 Hz (high cutoff frequency from 35 to 525 Hz) and filtered the microphone signals. As the sampling frequency was 2,560 Hz, this results in an image of 50 by 1,024 for a segment of 0.4 s. This image has a finer frequency resolution, and the phase information is preserved. In what follows, this type of image is referred to as the band-pass-filtered signal stack.

**Figure 3:**
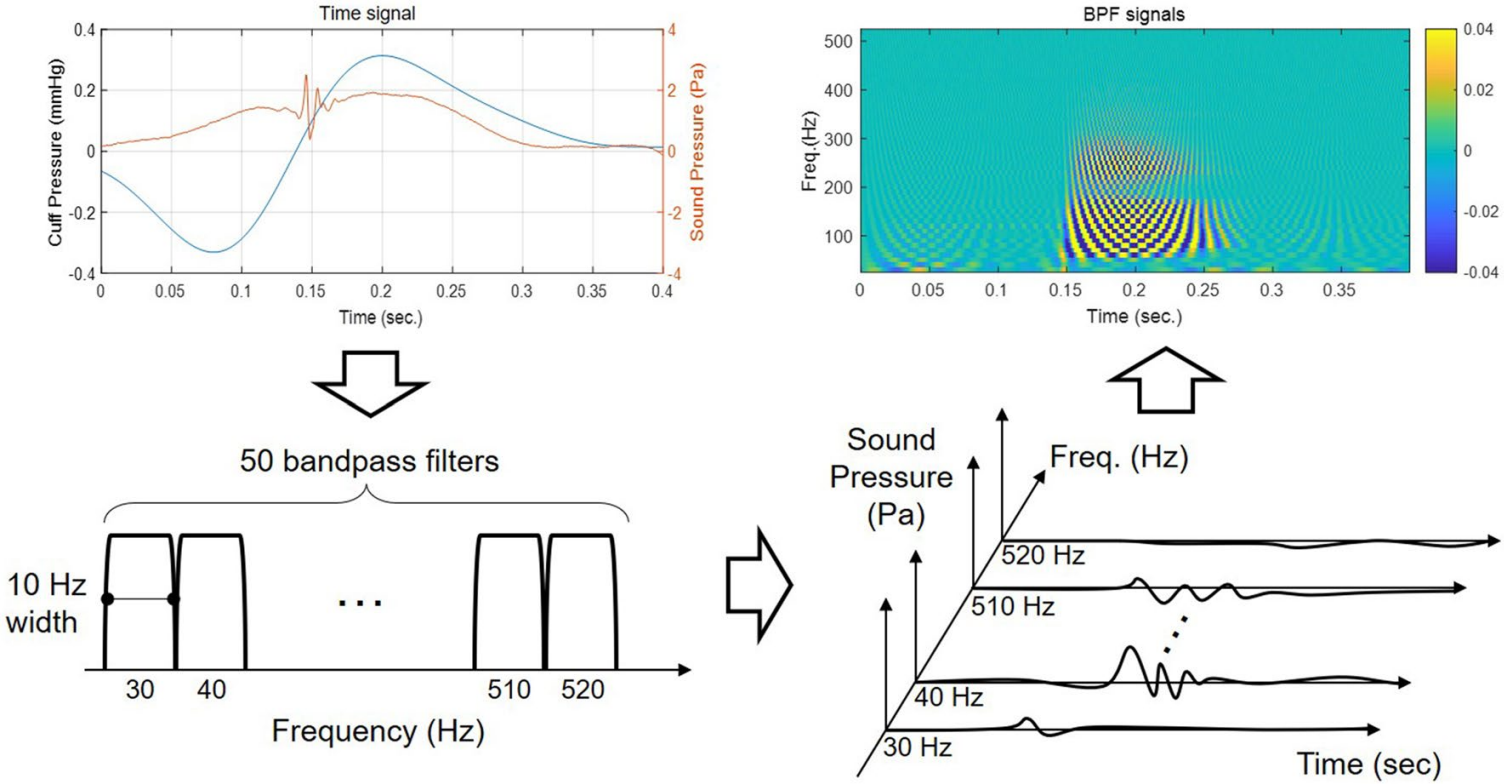
An example of a time signal (top left), the band pass filters (bottom left), the band-pass-filtered signals in waveforms (bottom right) and the band-pass-filtered signal stack in color (top right).

Figure 3 demonstrates a microphone signal and its images by band-pass filtering. Figure 3 (top) shows a microphone signal together with the cuff pressure oscillation for peak extraction. Figure 3 (bottom) shows the band-pass-filtered signal stack. This image was sent to the CNN model as the input image.

### Human-response-based labeling

**Figure 4:**
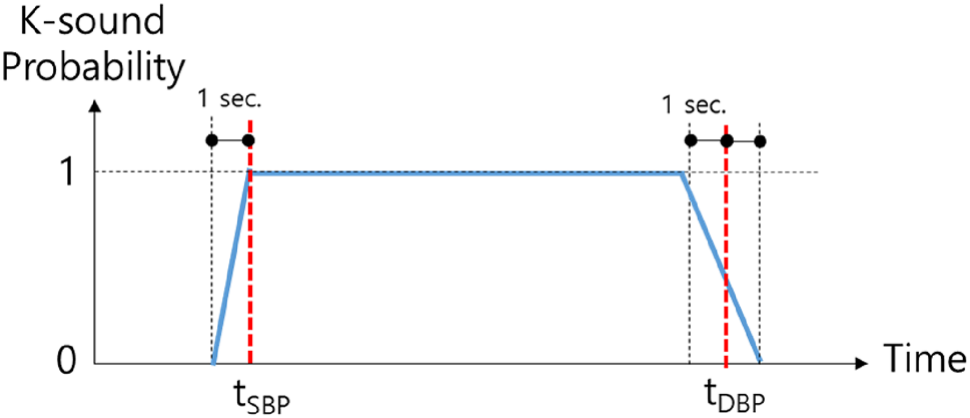
Determining probability labels.

**Figure 5:**
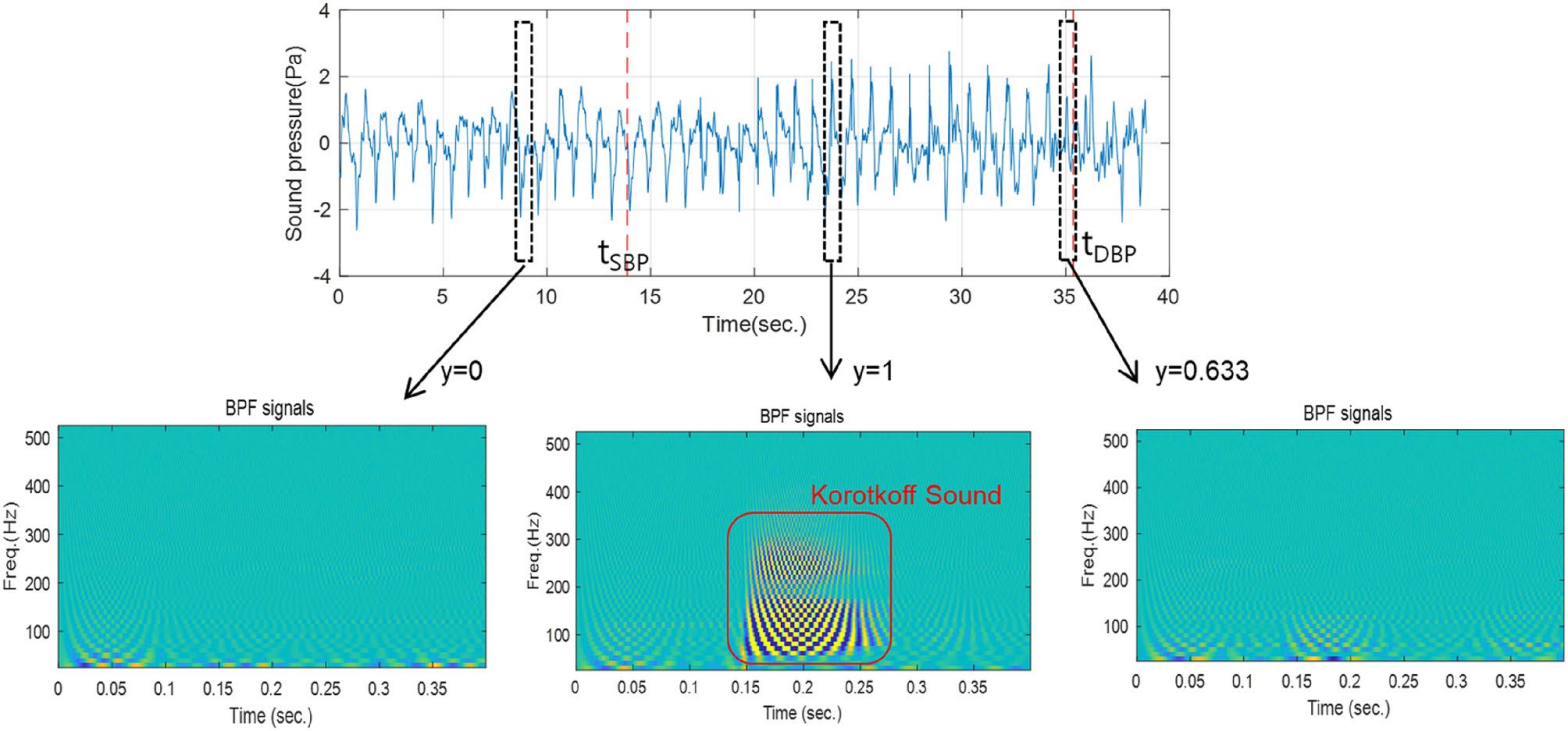
Examples of band-pass-filtered signal stacks and labels.

In binary classification problems, two labels, 0 and 1, are assigned to input signals. For example, in earlier work^12^, if a segment in a microphone signal contains K-sounds, 1 is assigned as the label, and if not, 0 is assigned. However, during the systole and diastole phases, there are substantial variations of the K-sounds^20^. Moreover, human subjects can respond late or early when deciding on the SBP and the DBP. To take this effect into account, we assumed that the distribution of K-sound probability would be 1 at the between SBP and DBP and 0 at the outside. Near SBP and DBP, the probability would change linearly as shown in Fig. 4. We also assumed that subjects could respond up to one second late for the SBP in general, while responding both early or late up to 1 s for the DBP. That is, a probability curve y is

1 $$y(t,{t}_{SBP},{t}_{DBP})=\left\{\begin{array}{l}0, \, t<{t}_{SBP}\text{-1 or }t>{t}_{DBP}+1\\ 1, \, {t}_{SBP}<t<{t}_{DBP}-1\\ t-\left({t}_{SBP}-1\right), \, {t}_{SBP}-1<t<{t}_{SBP}\\ -0.5\left(t-\left({t}_{DBP}+1\right)\right), \, {t}_{DBP}-1<t<{t}_{DBP}+1\end{array}\right.$$

where *t*_SBP_ and *t*_DBP_ are the time instances when the SBP and the DBP were determined, respectively. This probability curve has not been proved to be optimal, but finding the best fitting probability curve is out of the scope of this study. Figure 5 presents examples of input signals and the corresponding labels. The first and the second pulses have values of 0 and 1, respectively, but the third pulse has a value in between those two, at 0.633.

### CNN model and augmentation

A typical CNN model called Resnet 18 is utilized in this study^25^. To adapt this model to the present task, a layer that converts the number of output features to one was added to the last layer, and the first convolution layer was changed so that the input size would be equal to that of the band-pass-filtered signal stacks. A sigmoid activation function was applied to the output. The model is trained such that the binary cross entropy in Eq. (2) is reduced, as shown below:

2 $$L\left(y,\widehat{y}\right)=-\frac{1}{N}{\sum }_{n=1}^{N}\left[{y}_{n}\mathrm{log}{\widehat{y}}_{n}+\left(1-{y}_{n}\right)\mathrm{log}\left(1-{\widehat{y}}_{n}\right)\right]$$

Here ${y}_{n}$ is the true value and ${\widehat{\mathrm{y}}}_{n}$ is the predicted value for the $\mathrm{n}$ th input. When ${\widehat{\mathrm{y}}}_{n}$ is either 0 or 1, during the training, the mean squared error between the output value and the true value is monitored:

3 $$\overline{e} ^{2} = \frac{1}{N}\sum _{{n = 1}}^{N} \left( {y_{n} - \hat{y}_{n} } \right)^{2}$$

Data augmentation is known to be effective to prevent neural networks from overfitting^26–29^. We added white noise to the input images and applied a circular shift along the time axis as well as a cutout^28^ during the training. The amplitude of the white noise, randomly chosen, was in range of 0–0.1 of the root mean squared value of the input signal to prevent the DNN from overfitting. The time shift was also randomly chosen in the range of − 97.7 to 97.7 ms. For the cutout, a rectangular region was selected in the input, and values were replaced by a value of 0.05. The size of the region was 30% of the input, 15 by 307. The position of the region was randomly selected.

### Decision rule of BPs

**Figure 6:**
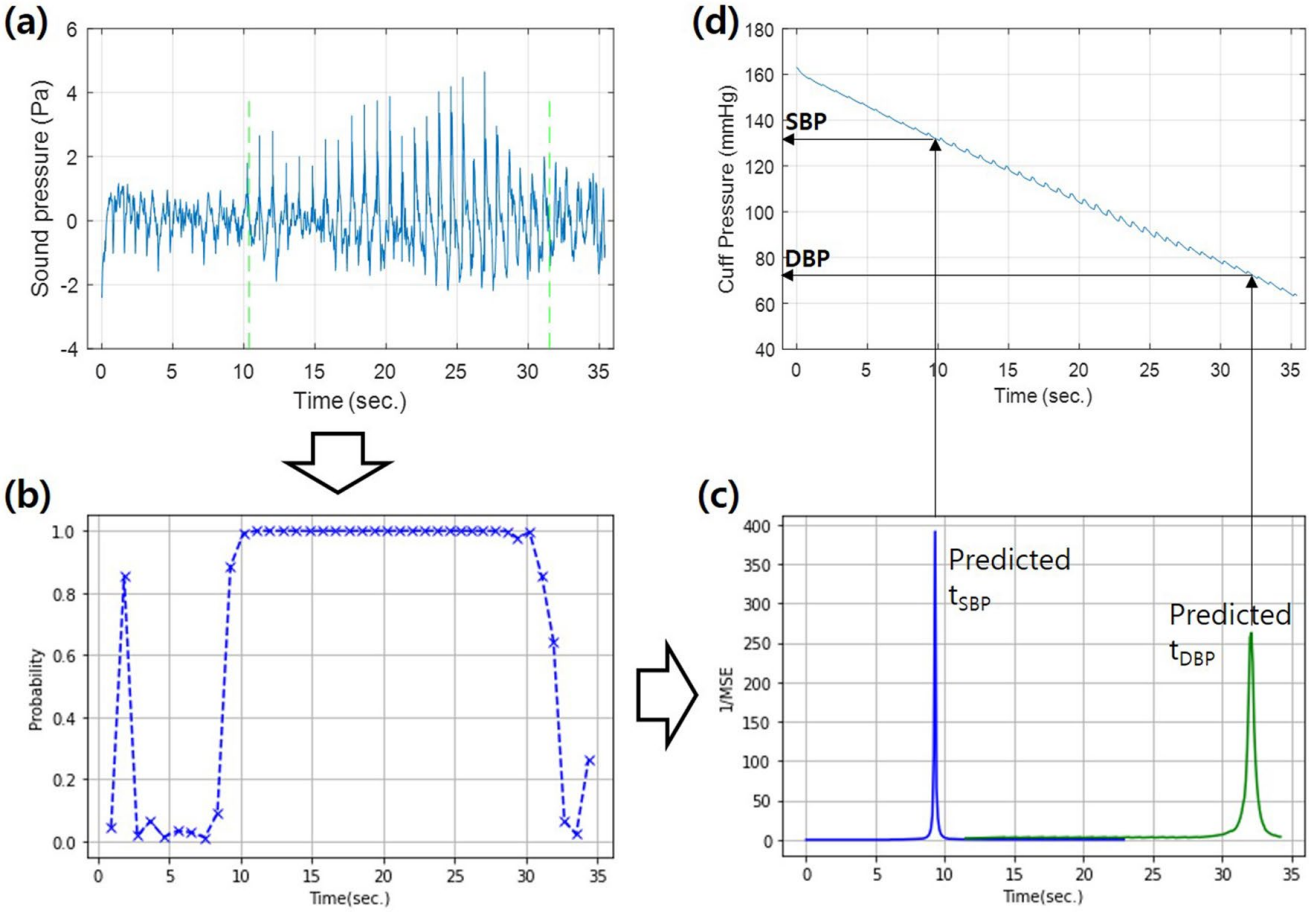
Blood pressure decision: (**a**) sound signal, (**b**) K-sound probability values, (**c**) prediction of *t*_SBP_ and *t*_DBP_, and (**d**) SBP and DBP decisions.

To compute the SBP and the DBP from sound signal (Fig. 6a), output probability values were arranged in the time domain, as shown in Fig. 6b. These values are denoted as ${y(t=t}_{n})$ where *n* is the index indicating the order of the signals, *n* = 1 to *N*. Subsequently, by comparing these values with the probability label curve in Fig. 4, *t*_SBP_ and *t*_DBP_ can be calculated. To estimate *t*_SBP_, a value *t*_n_ is first assumed to be equal to *t*_SBP_, and ten consecutive values of the label curve around *t*_SBP_, *y*(*t* = *t*_n-5_, *t*_SBP_ = *t*_n_, *t*_DBP_), *y*(*t* = *t*_n-4_, *t*_SBP_ = *t*_n_, *t*_DBP_), …, y(*t* = *t*_n+4_, *t*_SBP_ = *t*_n_, *t*_DBP_) are obtained. Then, the mean squared error (MSE) for the SBP is calculated as

4 $$\overline{\varepsilon }_{SBP}^{2} \left[ n \right] = \frac{1}{10}\sum\limits_{{n^{\prime} = - 5}}^{4} {\left\{ {\hat{y}_{{n + n^{\prime}}} - y\left( {t = t_{{n + n^{\prime}}} ,t_{SBP} = t_{n} ,t_{DBP} = t_{N} } \right)} \right\}^{2} }$$

That is, the predicted probability values are compared only with the onset part of the label curve. Note that *t*_DBP_ was set to be *t*_N_ not to affect the MSE value. When n + n′ is smaller than 1, $\widehat{{\text{y}}}_{{n + n^{\prime}}}$ is not defined, and thus excluded in the calculation. Likewise, the mean squared error for the DBP is

5 $$\overline{\varepsilon }_{DBP}^{2} \left[ n \right] = \sum\limits_{{n^{\prime} = - 5}}^{4} {\left\{ {\hat{y}_{{n + n^{\prime}}} - y\left( {t = t_{{n + n^{\prime}}} ,t_{SBP} = t_{1} ,t_{DBP} = t_{n} } \right)} \right\}^{2} }$$

where *t*_SBP_, which was set to be *t*_1_, does not affect this MSE value. The probability values were compared only with the offset part of the label curve.

Figure 6c shows the inversion of the MSE values, $1/\overline{\varepsilon }_{SBP}^{2} \left[ n \right]$ and $1/\overline{\varepsilon }_{DBP}^{2} \left[ n \right]$. The point *t*_n_ where the MSE is the smallest is determined as *t*_SBP_ (the maximum in Fig. 6c. This process is repeated for all possible *t*_DBP_ values. The cuff pressure values at these time instances, *t*_SBP_ and *t*_DBP_, are obtained as the SBP and the DBP, respectively, as shown in Fig. 6d).

This decision method was validated with the training set. Although the mean error and the standard deviation of the SBP and the DBP were small, it was found nonetheless that the performance can be improved further by thresholding the probability values by 0.9. That is, the predicted probability values that exceed 0.9 were replaced with 0.9, and the label curve was multiplied by 0.9.

6 $$\hat{y}_{n} = \left\{ {\begin{array}{*{20}c} {\hat{y}_{n} , \, \hat{y}_{n} < 0.9} \\ {0.9, \, \hat{y}_{n} \ge 0.9} \\ \end{array} } \right.$$

## Results

### Baseline method

**Figure 7:**
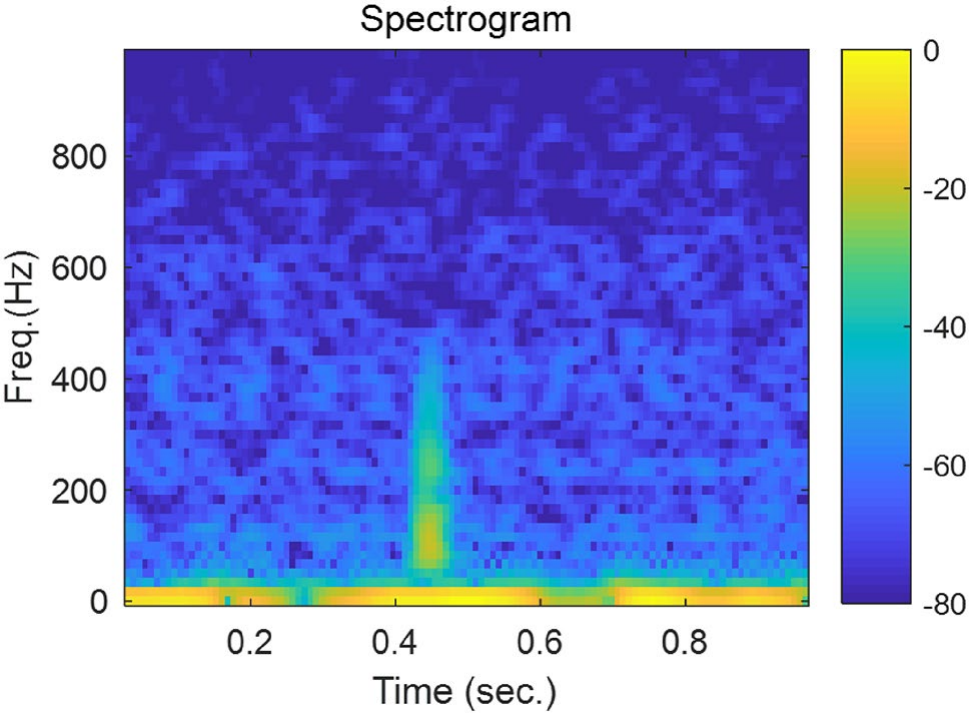
Input spectrogram for the baseline method of the signal shown in Fig. 3.

**Figure 8:**
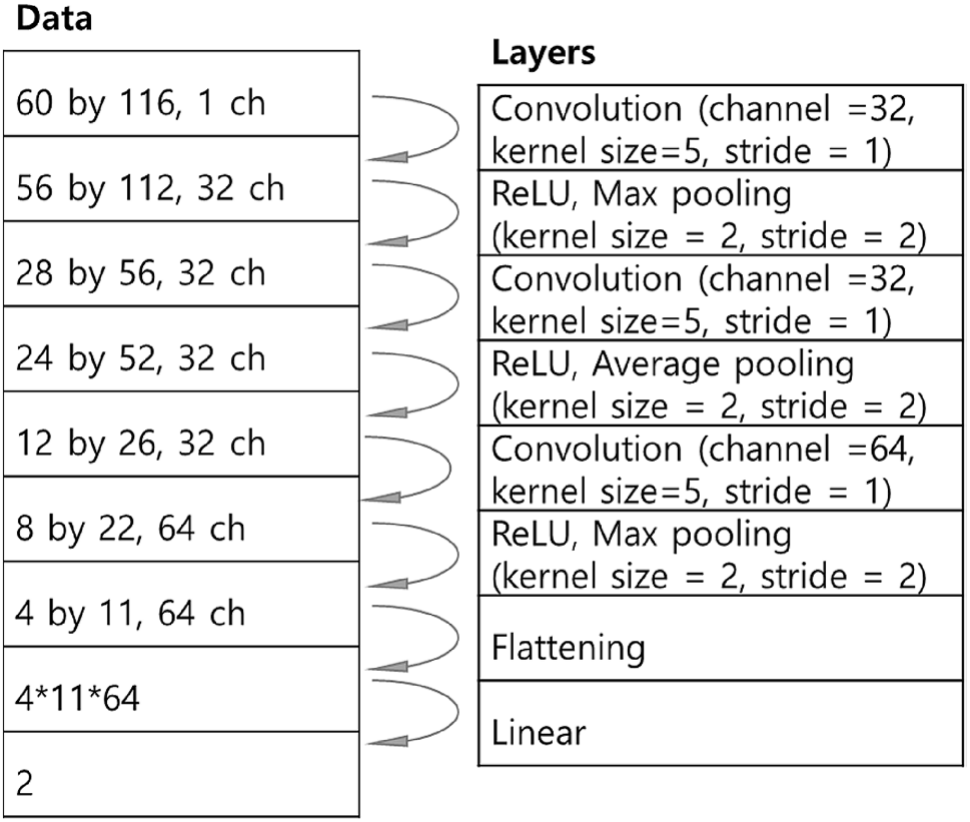
Structure of the CNN model of the baseline method.

A simple CNN method similar to what was used in earlier work^12^ is employed here as the baseline method. After resampling at 2 kHz, a time segment of one second was converted into the magnitude of a spectrogram with 87% overlap. The length of a frame was 60 ms, which leads to 16.7 Hz for the frequency resolution. The size of the input was 60 by 116 and an example image is shown in Fig. 7. The magnitude is expressed on the dB scale, where the reference value was 1 Pa. The same CNN model was designed as shown in Fig. 8. Although not stated in the aforementioned study^10^, the ReLU activation function^13^ was applied before max pooling and average pooling because it improved the performance.

The random search strategy was applied to find the best combination of parameters^30^. Three parameters, the learning rate, the batch size, and the weight decay, were randomly chosen to build 200 parameter sets. The learning rate was chosen in the range of [10^–5^ to 10^–2^], the chosen batch size was in the range of^25,28^, and the range for the weight decay was [10^–6^, 10^–3^]. When the validation accuracy increased, the model was saved. When there is no improvement in the validation accuracy during 100 epochs, the learning stopped, and the model was considered to be the best with the given parameter set.

Among the 200 best models, the best model was that with the highest validation accuracy, 92.9%. For this model, the test accuracy rate was 93.5%. The SBP and the DBP were determined in the same manner used in the aforementioned study^12^: when at least two consecutive beats are identified as K-sounds, the cuff pressure at the moment of the first beat is the SBP, and when beats no longer appear, the cuff pressure at that moment is the DBP.

**Figure 9:**
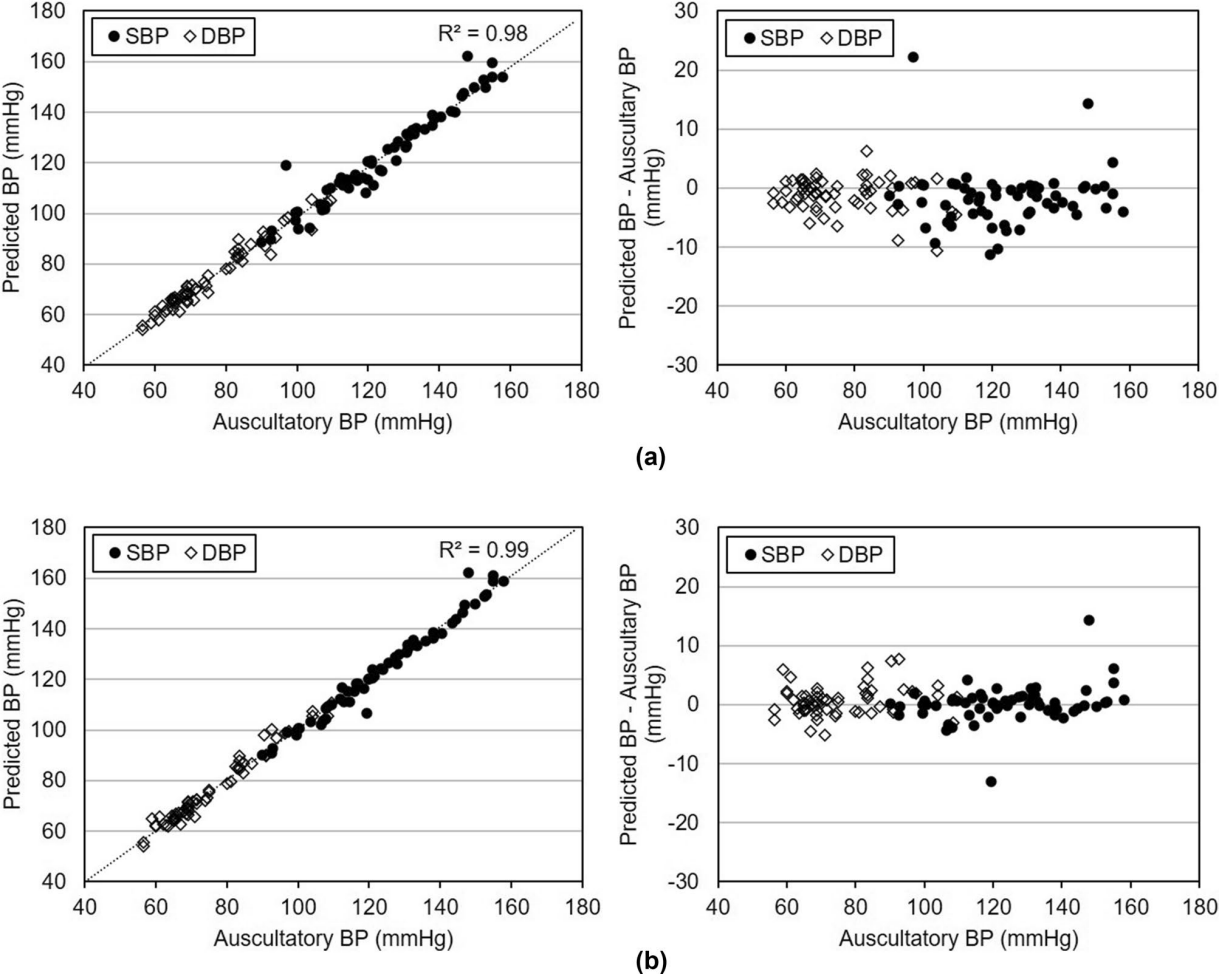
Predicted SBP and DBP: (**a**) baseline method; (**b**) proposed method.

The mean error + standard deviation (baseline BP – auscultatory BP) was − 1.7 ± 4.9 mmHg for the SBP and − 1.2 ± 2.8 mmHg for the DBP. The baseline model performed more accurately than the oscillometric method. Figure 9a shows the measured SBPs and DBPs with this method.

### Proposed method

The same random search strategy was applied to find the best model with the proposed method. The same ranges of the parameters were assigned, and 200 parameter sets were chosen. Instead of 100 epochs, when there is no improvement for 50 epochs, learning stopped because it took more time for processing otherwise. In addition, the validation accuracy was replaced by the validation MSE to select the best model. That is, among the 200 best models, the best model chosen was that with the smallest validation MSE, which was 0.039 in this case. With this model, the test MSE was 0.035.

In the proposed method, SBP and DBP were determined according to the decision rule described in Section III. As shown in Fig. 9b, the mean and the standard deviation (proposed BP – auscultatory BP) were 0.2 ± 3.1 mmHg for the SBP and 0.7 ± 2.5 mmHg for the DBP.

Compared to results of the baseline model, the proposed model has a smaller mean and a smaller standard deviation as summarized in Table 3. With the baseline model, twelve samples had SBP errors exceeding 5 mmHg, while six samples had DBP errors that exceeded 5 mmHg. In contrast, with the proposed model, only three and five samples had SBP and DBP errors that exceeded 5 mmHg.

**Table 3:** Comparison between baseline and proposed methods.

Method	SBP		DBP	
	Average ± standard deviation (mmHg)	Number of case exceeding 5 mmHg error	Average ± standard deviation (mmHg)	Number of case exceeding 5 mmHg error
Baseline method	− 1.7 ± 4.9	12	− 1.2 ± 2.8	6
Propose method	0.2 ± 3.1	3	0.7 ± 2.5	5

## Discussion

Since we used practical signals, it was not possible to control the signal-to-noise ratio (SNR). Instead, the SNR values can be approximately calculated. The noise level can be estimated as the root-mean-square (RMS) values of the signal before *t*_SBP_, and the signal level can be estimated as the RMS of the peak values between *t*_SBP_ and *t*_DBP_. We obtained the SNR values, and the mean was 7.93 dB with the standard deviation of 5.27 dB. The minimum was − 2.9 dB. and the maximum was 18.5 dB. For example, the SNR of the sound signal shown in Fig. 5 was calculated as 8.2 dB.

We calculated the SBP and the DBP errors for 26 sound signals that have lower SNR values than the mean value. The results were shown in Table 4. The prediction errors with the proposed methods decrease for the low SNR signals, and the number of cases exceeding 5 mmHg error was 0 both for the SBP and the DBP.

**Table 4:** Prediction errors for low SNR signals.

Method	SBP		DBP	
	Average ± standard deviation (mmHg)	Number of case exceeding 5 mmHg error	Average ± standard deviation (mmHg)	Number of case exceeding 5 mmHg error
Baseline method	1.4 ± 5.7	7	0.3 ± 2.0	1
Propose method	− 0.1 ± 2.0	0	− 0.3 ± 1.8	0

**Figure 10:**
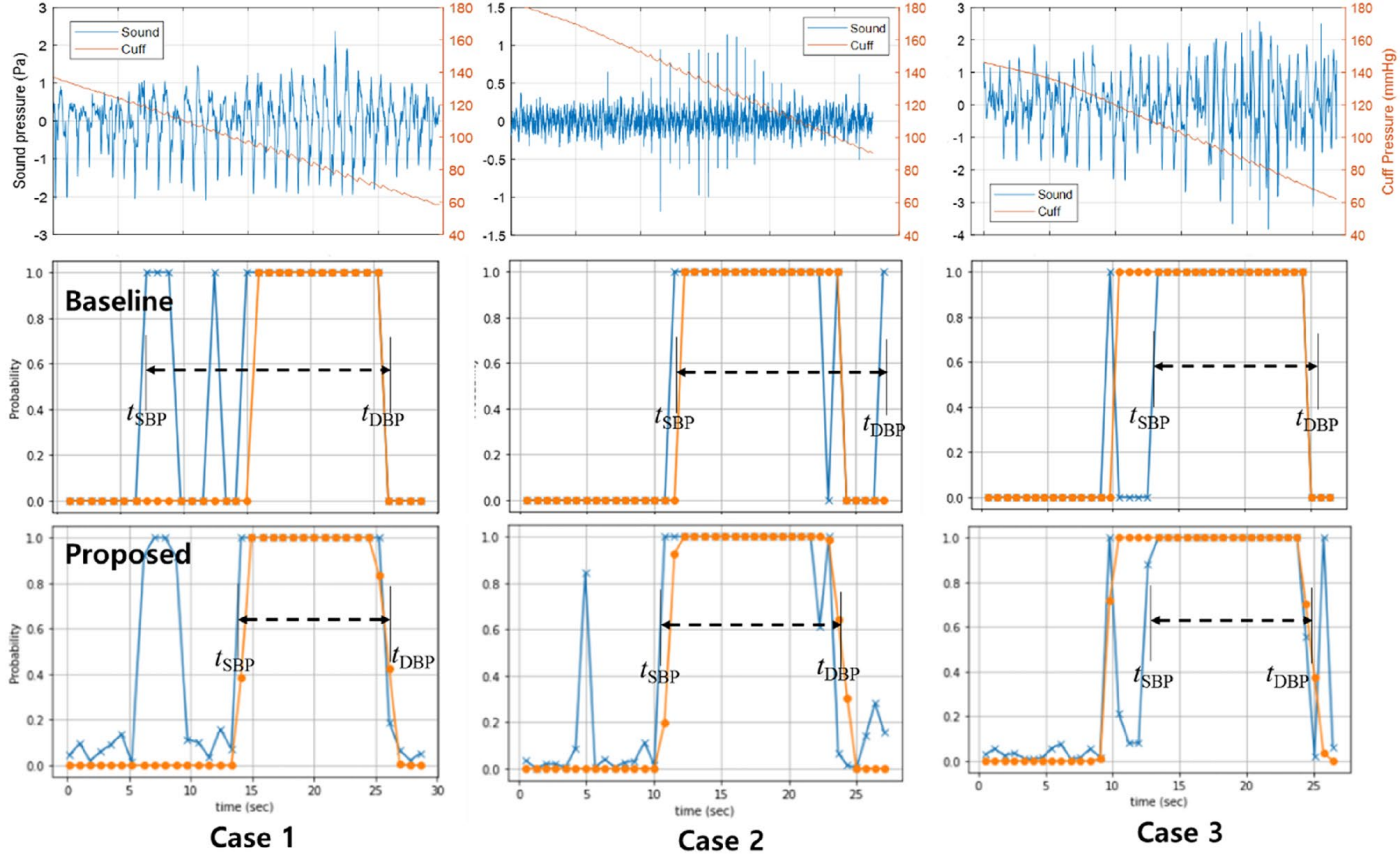
Sound signal (top) and predicted probability with the baseline (middle) and the proposed model (bottom) in three noisy cases.

To analyze the results in a qualitative way, we observed three cases as shown in Fig. 10. The SNR was 7.43, 16.4, and 14.8 dB, respectively. The first row shows sound signals. The second row shows the probability predicted by the baseline model (‘x’) with the true values (‘●’) by the auscultatory method. The predicted range of the K-sounds is indicated by the dashed arrow line. The third row is that by the proposed model.

In the first case, the SNR of the first case was below the average, and the true BPs are 97 and 67.5 mmHg correspondingly for the SBP and the DBP. The baseline model has − 22.11 mmHg for the SBP error because three consecutive values that occurred before *t*_SBP_ mislead. In contrast, the proposed model has only − 1.91 mmHg for the SBP error in spite of four consecutive high values before the *t*_SBP_. This was possible by the decision rule based on the MSE.

In the second case, the true BPs are respectively 147 and 104 mmHg for the SBP and the DBP, and the baseline model has 10.58 for the DBP error because a peak occurred after *t*_DBP_. In contrast, the proposed model has 2.00 mmHg for the DBP error because the probabilities were small after *t*_DBP_, and only one peak does not affect the decision. Even if the peak after the *t*_DBP_ has a high probability value, the MSE must have been large, and consequently the peak could not have affected the decision.

These two cases imply that the decision rule of the baseline method can be sensitive to external noise signals, whereas the proposed method is more immune to these signals.

Nonetheless, both methods failed in the third case in terms of the SBP errors that exceed 10 mmHg. Even after the systolic pulse, some pulses were not assessed as K-sounds, and the SBP was determined to be lower than the true value. This issue was also reported earlier^12^ as the auscultatory gap. After the first systolic pulse, a few next pulses have occasionally small amplitudes, and are almost inaudible. To overcome this issue, it would be helpful to add relatively noisier data to the training set so that the CNN model can detect K-sounds even with the small amplitudes.

## Conclusion

In this work, a CNN model that automatically identifies audible Korotkoff sounds associated with cuff pressure oscillation was proposed. To improve the performance, a labeling curve was proposed based on the human response that provides the probability of each pulse in the training set and determines the blood pressure for the test set. In addition, the band-pass-filtered signal stacks were used as the input images.

The results were evaluated with a test set that contains noisy data. Compared to the baseline model, the proposed model achieved more precise BP prediction results in terms of the mean and standard deviation values of the error from BP values measured by the auscultatory method. With larger amounts of various types of data, the proposed method can potentially achieve more precise and robust results.
